# *n-i-p* Nanocrystalline Hydrogenated Silicon Solar Cells with RF-Magnetron Sputtered Absorbers

**DOI:** 10.3390/ma12101699

**Published:** 2019-05-25

**Authors:** Dipendra Adhikari, Maxwell M. Junda, Corey R. Grice, Sylvain X. Marsillac, Robert W. Collins, Nikolas J. Podraza

**Affiliations:** 1 Department of Physics & Astronomy and The Wright Center for Photovoltaics Innovation & Commercialization, University of Toledo, Toledo, OH 43606, USA; Dipendra.Adhikari@rockets.utoledo.edu (D.A.); Maxwell.Junda@utoledo.edu (M.M.J.); Corey.Grice@utoledo.edu (C.R.G.); Robert.Collins@utoledo.edu (R.W.C.); 2 Virginia Institute of Photovoltaics, Old Dominion University, Norfolk, VA 23529, USA; Smarsill@odu.edu

**Keywords:** Hydrogenated nanocrystalline silicon, Real time spectroscopic ellipsometry, Dielectric function, RF-sputtering, PECVD, nc-Si:H *n-i-p* solar cell

## Abstract

Nanocrystalline hydrogenated silicon (nc-Si:H) substrate configuration *n-i-p* solar cells have been fabricated on soda lime glass substrates with active absorber layers prepared by plasma enhanced chemical vapor deposition (PECVD) and radio frequency magnetron sputtering. The cells with nanocrystalline PECVD absorbers and an untextured back reflector serve as a baseline for comparison and have power conversion efficiency near 6%. By comparison, cells with sputtered absorbers achieved efficiencies of about 1%. Simulations of external quantum efficiency (EQE) are compared to experimental EQE to determine a carrier collection probability gradient with depth for the device with the sputtered *i*-layer absorber. This incomplete collection of carriers generated in the absorber is most pronounced in material near the *n/i* interface and is attributed to breaking vacuum between deposition of layers for the sputtered absorbers, possible low electronic quality of the nc-Si:H sputtered absorber, and damage at the *n/i* interface by over-deposition of the sputtered *i*-layer during device fabrication.

## 1. Introduction

Hydrogenated silicon (Si:H) is an important and widely studied material for thin film photovoltaic (PV) applications because it is non-toxic, inexpensive, and earth abundant. Within the solar irradiance spectrum of interest for most solar cells, hydrogenated amorphous silicon (a-Si:H) shows a higher absorption coefficient compared to indirect band gap nanocrystalline silicon (nc-Si:H) and single crystal silicon (c-Si) absorbers. Comparatively thin (~0.3 µm) a-Si:H, moderately thick (~1.8 µm) nc-Si:H, and much thicker (~200 µm) c-Si absorber layers are required for sufficient absorption of incident solar radiation. Among these two thin film absorbers, a-Si:H is vulnerable to the Staebler–Wronski effect [1] resulting in degradation under illumination, whereas nc-Si:H suffers from less or no light induced degradation. Several techniques are available for the fabrication of Si:H thin films including plasma enhanced chemical vapor deposition (PECVD: RF and VHF) [2,3,4,5,6,7,8], RF magnetron sputtering [9,10,11,12,13], hot-wire chemical vapor deposition [14,15,16], and other technologies [17,18]. Among these techniques, Si:H PV devices with PECVD absorbers are the most widely studied [2,3,4,5,6,7,8], but require toxic silicon carrying precursor gases (SiH_4_, Si_2_H_6_). By contrast, there is comparatively sparse literature describing sputtered Si:H, and nc-Si:H in particular [11,19], but the sputtering process from a solid silicon target in reactive hydrogen is completely nontoxic and fairly simple. In addition, sputtering [9] has been shown to provide effective control over many microstructural properties of the resultant films including crystallinity, hydrogen incorporation, and surface morphology, all of which are of interest in the various applications of thin Si:H films in PV and other devices. Sputter deposition also enables *p*- and *n*-type dopant atoms to be introduced in silicon targets, eliminating toxic dopant gases needed in PECVD [20]. Solid silicon targets have also been used in pulsed laser deposition of Si:H [21]. Sputtering, however, is a demonstrated industrially scalable process already used for large area depositions needed for applications like PV [22], making it a strong potential alternative to PECVD in manufacturing. Here we have demonstrated working PV devices produced with sputtered nc-Si:H absorber layers and compare overall device performance to those produced with the more conventional PECVD absorbers. Although the PV performance of these devices can be improved, there is potential for development of nc-Si:H without the need for toxic source gases to reduce overall device fabrication cost. We report here one of the only studies of functioning solar cells incorporating sputtered nc-Si:H absorber layers.

## 2. Experimental Details 

**Figure 1:**
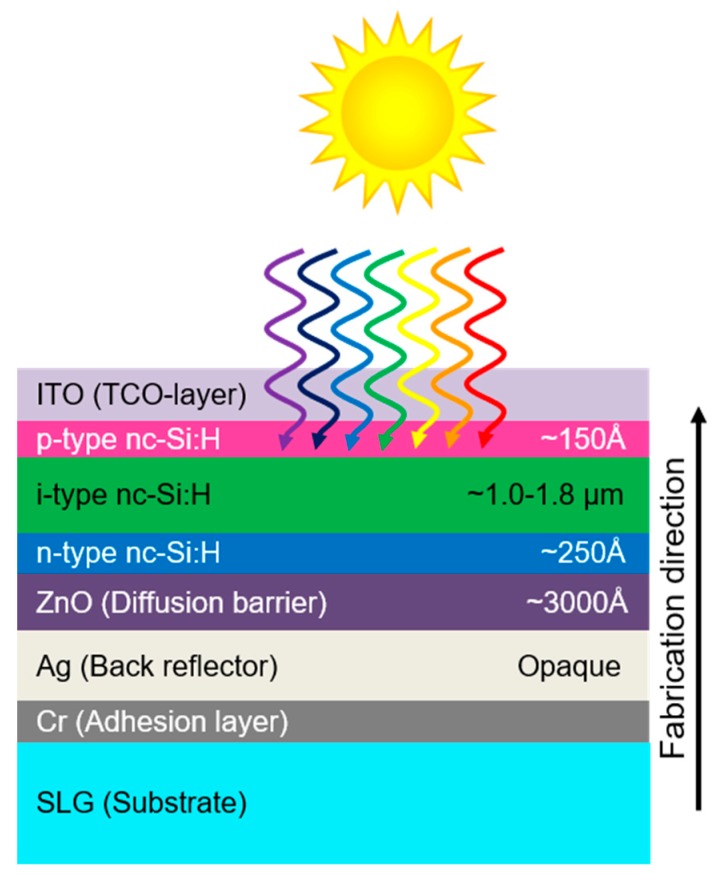
Schematic diagram of nc-Si:H absorber based solar cell in the *n-i-p* substrate configuration.

The general layered structure for the *n-i-p* configuration devices studied is a soda lime glass (SLG) supporting substrate, Cr adhesion layer, Ag metal back reflector, ZnO transparent conducting oxide diffusion barrier, nc-Si:H *n*-layer, nc-Si:H *i*-layer, nc-Si:H *p*-layer, and indium tin oxide (ITO) transparent conducting front dot contact defining the area of the cells. This structure is depicted schematically in Figure 1. The sputtered *i*-layers of primary interest to this work are prepared in a standalone sputter chamber (AXXIS^TM^, K. J. Lesker Co.), while all other layers are prepared in a multi-chamber, load locked cluster tool fully under vacuum (MV Systems). Thus, for cells prepared with sputtered *i*-layers, vacuum break and exposure to atmosphere occurs both immediately before and after the *i*-layer deposition. Three types of these devices are constructed with differently fabricated nc-Si:H *i*-layers, including (1) sputtered nc-Si:H layers, (2) PECVD nc-Si:H *i*-layers where the devices were removed from vacuum into laboratory atmosphere both immediately prior to and after the *i*-layer deposition, and (3) otherwise identical PECVD nc-Si:H *i*-layers incorporated into devices fully prepared in the cluster tool without leaving vacuum. These three sample configurations provide the opportunity to compare the devices with sputtered *i*-layers to those deposited with PECVD that have the same vacuum breaks (2) and a control sample fabricated without breaking vacuum (3).

SLG substrates (Pilkington North America) are cleaned with detergent (Micro-90, International Products Corp.) in a heated ultrasonic bath, rinsed several times with deionized water, and then dried with pure N_2_ gas in air before thin film deposition. The Cr, Ag, ZnO, and ITO layers are all deposited via RF magnetron sputtering in the cluster tool. The PECVD nc-Si:H *n*-, *i*-, and *p*-layers are deposited at radio frequency (13.56 MHz) using a H_2_ diluted SiH_4_ precursor gas mixture described by the dilution ratio *R* = [H_2_] / [SiH_4_]. The dopant gas ratios for *n*- (*D* = [PH_3_]/[SiH_4_]) and *p*-layers (*D* = [B_2_H_6_]/[SiH_4_]), which can have considerable influence on the structural and electronic properties, are fixed at *D* = 0.0125. The chamber base pressure is ~10^−7^ Torr before each deposition. The substrate temperature, deposition pressure, plasma RF power density, and gas flows are listed for each layer in Table 1. It is worth noting that nanocrystallinity arising from these conditions in *n*-, *i*-, and *p*-layers is confirmed via in-situ real-time spectroscopic ellipsometry (RTSE). In-situ RTSE measurements have been performed over the near infrared to ultraviolet spectral range of 0.74–5.90 eV at a 70° angle of incidence using a chamber-mounted dual rotating compensator multichannel ellipsometer (RC2, J.A. Woollam Co., Inc.) [23]. Modeling of the RTSE data allows for the determination of the thickness, surface roughness, and nanocrystalline volume fraction of each film. A set of *p*-, *i*-, and *n*-layers are deposited as a function of *R* and monitored by RTSE to identify the appropriate *R* for nanocrystalline phase growth beginning immediately at the start of deposition [3,4,9]. It has been observed that the highest electronic quality nc-Si:H films are produced with the lowest value of *R* at which the nanocrystalline phase dominates growth [24,25]. Thus, each film is deposited with the minimum possible *R* that will still produce nanocrystalline growth from the start of deposition. It is determined that these minimum values of *R* required for immediate nanocrystalline growth for the *n*-, *i*-, and *p*-layers are *R* = 100, 125, and 250, respectively. The deposition rate of the PECVD nc-Si:H *i*-layer deposited on the top of a nc-Si:H *n*-layer is about 0.6 Å/s.

For the fabrication of sputtered *i*-layers, an undoped 7.62 cm diameter Si target (99.999% purity, K.J. Lesker Co.) spaced 13.5 cm from the substrate is sputtered using RF power of 250 W in a controllable H_2_ + Ar environment with a substrate temperature of 200 °C. Sputtering is performed at 10 mTorr gas pressure with a constant hydrogen-to-argon gas flow ratio, *p_H2_* = 100% × [H_2_]/{[H_2_] + [Ar]} in upstream mode for each deposition. Previous work [9,10] has successfully optimized these deposition conditions to produce reasonable growth rate material with controllable microstructure as identified by RTSE. Microstructure during sputtering is controlled by *p_H2_* and typical growth rates are approximately 1.3 Å/s. 

The *n-i-p* devices with PECVD *i*-layers both exposed and not exposed to air are fabricated on 15.24 cm × 15.24 cm glass substrates with 256 dot cells each having an active area of 0.0707 cm^2^. Devices incorporating sputtered nc-Si:H *i*-layers deposited with different *p_H2_* (80, 85, and 90%) are fabricated, and results for *p_H2_* = 90% sputtered layers are included here. Because of the complexity of using larger substrates in the stand alone sputtering chamber, *n-i-p* devices with sputtered nc-Si:H *i*-layers are fabricated on 5.08 cm × 5.08 cm glass substrates resulting in 25 dot solar cells. Photocurrent density versus voltage (J-V) characteristics are measured under simulated AM1.5G (100 mW/cm^2^) illumination from a 450 W Xenon light source (Oriel, Model 9119, Newport) with a digital source meter (Keithley 2440) in air at room temperature. Dark current is also measured using the same system. External quantum efficiency (EQE) measurements are performed (model IVQE8-C, PV Instruments) to characterize spectrally resolved device performance. Topography and roughness measurements of the Cr/Ag/ZnO back reflector (BR) structure are also performed using an atomic force microscope (AFM) operated in tapping mode (Nanoscope V, Veeco). Ex-situ spectroscopic ellipsometry measurements of the BR structure are performed using a single rotating compensator multichannel ellipsometer (J.A. Woollam M-2000FI) [26]. EQE spectra of solar cells with *i*-layers fabricated by PECVD and sputtering are simulated by using complex dielectric function (*ε* = *ε*_1_ + i*ε*_2_) spectra and thicknesses of layers that are obtained using the same spectroscopic ellipsometry data analysis procedure explained in Ref. [4]. Spectra in *ε* for PECVD and sputtered *i*-layers are represented as Bruggeman effective medium approximations [27] of crystal silicon for simplicity [28] and a-Si:H with a band gap of 1.8 eV [29]. Initially during simulation, it is assumed that each photon absorbed in the *i*-layer generates an electron–hole pair able to be collected without recombination losses. To account for the incomplete collection of charge carriers generated in the sputtered *i*-layer, a collection probability profile is introduced into the simulation to identify regions of reduced carrier collection.

## 3. Results

**Figure 2:**
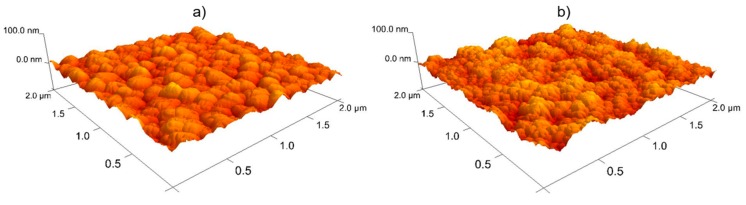
Three-dimensional atomic force micrographs (AFM) showing surface morphology of (**a**) Ag deposited on SLG/Cr and (**b**) ZnO deposited on SLG/Cr/Ag.

Before depositing nc-Si:H layers, the sputtered BR structure is prepared. To obtain surface roughness thickness of the BR with and without the ZnO diffusion barrier (Ag deposited on SLG/Cr and ZnO deposited on SLG/Cr/Ag substrate), room temperature ellipsometric spectra (in *N* = cos 2Ψ, *C* = sin 2Ψ cos*Δ*, *S* = sin 2Ψ sin *Δ*) have been collected ex-situ at 70° angle of incidence over a spectral range from 0.74 to 5.90 eV. Experimental ellipsometric spectra have been fit to an optical model exactly the same as described in Ref. [4]. From the analysis of ellipsometric spectra collected from a representative sample, the surface roughness thickness of the optically opaque Ag layer deposited on a SLG/Cr substrate is 10 ± 2 nm. The ZnO layer deposited on SLG/Cr/Ag substrate is found to have bulk layer and surface roughness thicknesses of 332 ± 3 nm and 11.3 ± 0.5 nm, respectively. The surface roughness and topography of both the SLG/Cr/Ag and full SLG/Cr/Ag/ZnO BR structures are also obtained using AFM, with the surface morphology shown in Figure 2. Using this technique, the root-mean squared (RMS) roughness of Ag and ZnO surfaces are found to be 6.8 and 9.4 nm, respectively. Previous reports comparing surface roughness from ellipsometry and RMS roughness from AFM have shown that ellipsometry determined roughness is about 1.5 times that from AFM [30,31]. Here, ellipsometry determined roughness is on average about 1.35 times the RMS value from AFM. These surface roughness thickness values indicate that the BR structures prepared with the processes described here are relatively smooth compared to intentionally textured BRs. In general, increasing the path length of light through the use of a rough, scattering BR is a common route to increasing current generated in and PV conversion efficiency of a-Si:H and nc-Si:H solar cells [32,33,34,35,36]. However, for the purposes of assessing the applicability of sputtered nc-Si:H *i*-layers, a relatively planar BR surface is sufficient for comparison among the three different *i*-layer fabrication configurations previously described. This configuration also avoids any cracking in nc-Si:H films sometimes arising from deposition onto textured BRs [34,36] and will enable the most direct assessment of the electronic device quality of differently prepared intrinsic layers. Analysis of ellipsometric spectra shows similar surface roughness values of 3.3 ± 1.7 nm and 2.7 ± 0.3 nm of the *p*-layer at the top of the devices fabricated with PECVD and sputtered nc-Si:H *i*-layers, respectively. Similar light scattering at the top surface of the devices and nc-Si:H growth morphology are expected for devices with both PECVD and sputtered absorbers.

Optimization of thin film PV relies on characterizing the optoelectronic and structural properties of each layer and correlating these properties with device performance. Nanocrystallite growth in Si:H from the amorphous matrix is promoted by using increased hydrogen dilution ratio, *R,* in PECVD [4] and hydrogen to total gas ratio, *p_H2_*, in RF magnetron sputtering [9]. Growth evolution diagrams have been developed and used to guide production of materials in nc-Si:H *n-i-p* devices. For the cells incorporating sputtered nc-Si:H *i*-layers, the growth evolution diagram in Ref. [9] is used as a guideline, to ensure that the material sputtered onto the underlying nc-Si:H *n*-layer will nucleate nanocrystallites immediately. Sputtered *i*-layers on *n*-type PECVD nc-Si:H with *p_H2_* = 80, 85, and 90% are all within the nanocrystalline growth regime according to the growth evolution diagram and RTSE monitoring of film growth, and separate full devices are fabricated with each of these *p_H2_*. The devices fabricated with nc-Si:H *i*-layers at *p_H2_* = 90% are found to result in the best performance. The optical response for the nc-Si:H film prepared at *p_H2_* = 90% had the highest amplitude features in spectra in *ε* indicating the highest optical density among the nc-Si:H films prepared at *p_H2_* = 80, 85, and 90% [9,10]. The *n-i-p* devices incorporating PECVD nc-Si:H *i*-layers prepared at *R* = 125, which is the minimum value of *R* required for immediate nanocrystalline growth for the *i*-layers deposited on the top of *n*-type PECVD nc-Si:H, have better device performance.

**Figure 3:**
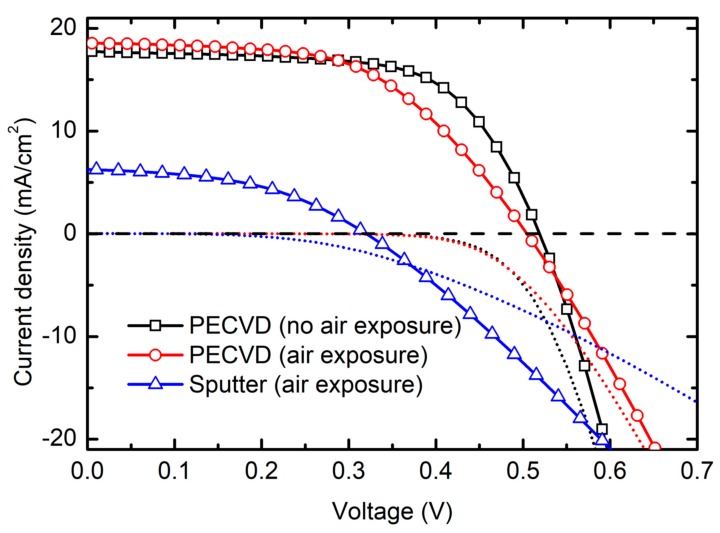
Current–voltage (J–V) measured under 100 mW/cm^2^ simulated AM1.5G irradiation and in the dark for the highest efficiency n-i-p devices incorporating a PECVD absorber without vacuum break (black squares, black dotted line), PECVD absorber with vacuum break before and after intrinsic layer deposition (red circles, red dotted line), and RF magnetron sputtered absorber also with vacuum break before and after intrinsic layer deposition (blue triangles, blue dotted line).

**Figure 4:**
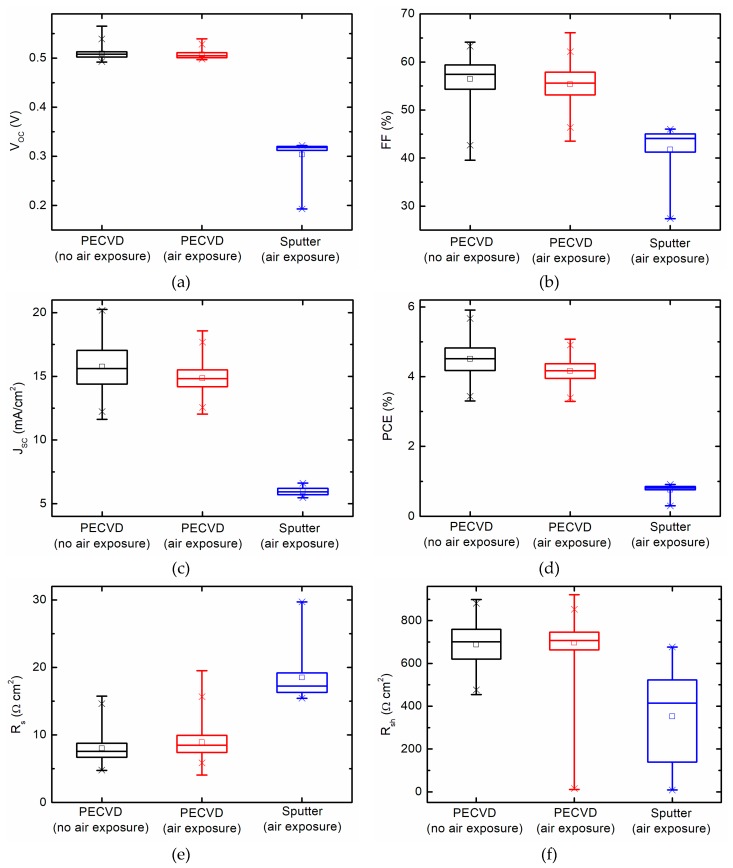
Solar cell performance parameters including open circuit voltage *V_OC_* (**a**), fill factor *FF* (**b**), short circuit current density *J_SC_* (**c**), power conversion efficiency *PCE* (**d**), series resistance *R_s_* (**e**), and shunt resistance *R_sh_* (**f**) for devices incorporating nc-Si:H absorbers prepared with different methods.

Figure 3 displays J–V characteristic curves for the highest efficiency solar cell of each of the three different *i*-layer preparation procedures measured under simulated AM1.5G illumination. The short circuit current (*J_SC_*), open circuit voltage (*V_OC_*), fill factor (*FF*), and power conversion efficiency (*PCE*) corresponding to devices plotted in Figure 3 are reported in Table 2. The device performance parameters presented in Table 2 are obtained for 1.8 µm thick PECVD absorbers and a 1 µm thick sputtered absorber. The box plot showing the comparison of device performance of all *n-i-p* nc-Si:H devices with different absorbers are shown in Figure 4. Figure 4 includes device performance parameters for all measured solar cells. For devices based on sputtered and PECVD *i*-layers fabricated with air exposure between nc-Si:H layers, substantially increased shunting is observed via the span of cell shunt resistance. This behavior is expected for thin film solar cell devices when vacuum is interrupted during fabrication and corresponds to lower device yield. Spatial non-uniformity of all deposited thin film layers, particularly the nc-Si:H absorber, are also sources of variation in device performance parameters.

As would be expected, the device with PECVD nc-Si:H *i*-layer fabricated without vacuum break is found to have highest *PCE* of 5.91%. By comparison, the cell incorporating a PECVD *i*-layer fabricated with vacuum breaks had *PCE* = 5.08%, and the device with a sputtered *i*-layer had 0.92%. For the device prepared with a PECVD nc-Si:H *i*-layer without breaking vacuum, *V_OC_* = 0.520 V and *FF* = 64.1% are consistent with values typically reported in literature [36,37]. *J_SC_* = 17.7 mA/cm^2^ is relatively low compared to the theoretical maximum based on the band gap of crystalline silicon and is attributed to the use of a planar non-textured back reflector and an absorber only 1.8 μm thick. This *J_SC_* value is consistent with other similarly designed devices [36,38,39]. 

The decreased efficiency of the device with the PECVD *i*-layer exposed to vacuum breaks relative to the device fabricated fully under vacuum is almost certainly due to oxidation and/or contamination of the interfaces on either side of the *i*-layer resulting from air exposure. The largest decrease in device performance parameters are in the *V_OC_* and *FF* which are reduced by 3% and 16%, respectively. This level of device performance reduction is expected considering a vacuum break during processing. Average *J_SC_* of the devices with *i*-layer exposed to air is lower compared to PECVD devices with absorbers prepared without air exposure (Figure 4). *J_SC_* of the highest efficiency cell with air exposure is slightly higher compared to corresponding highest efficiency cell without air exposure. This difference is likely the result of absorber layer thickness distribution due to spatial non-uniformity in each deposition and run-to-run variation between separate depositions. 

Compared to devices with PECVD *i*-layers, those with sputtered *i*-layers have substantially lower device performance parameters. The primary reason for worse performance when incorporating a sputtered absorber is because of lower electronic quality of the sputtered material itself or of interfaces with the *n*- and *p*-layers. We can see that vacuum breaks themselves only result in a decrease in efficiency of less than 1% so this does not account for the substantially lower efficiency of devices with sputtered *i*-layers. Average *V_OC_*, *J_SC_*, *FF*, and *PCE* of devices with a PECVD nc-Si:H absorber with no air exposure are slightly higher compared to PECVD nc-Si:H absorber with air exposure before and after deposition, while substantially lower mean device parameters are observed for sputtered absorber devices. For *n-i-p* nc-Si:H devices with sputtered absorber *i*-layers, higher series resistance (*R_s_*) and lower shunt resistance (*R_sh_*) are observed relative to devices with PECVD absorbers. The higher value of *R_s_* and lower value of *R_sh_* for devices with sputtered absorber results in lower *FF* and hence overall poor device performance compared to those with PECVD absorbers. The intersection between light and dark J–V curves for the example sputtered *i*-layer based device is more pronounced than those with PECVD absorbers. This behavior could arise due to lower electronic quality of sputtered bulk layer, interfaces (*n*/*i* and *p*/*i*), or both [40,41].

**Figure 5:**
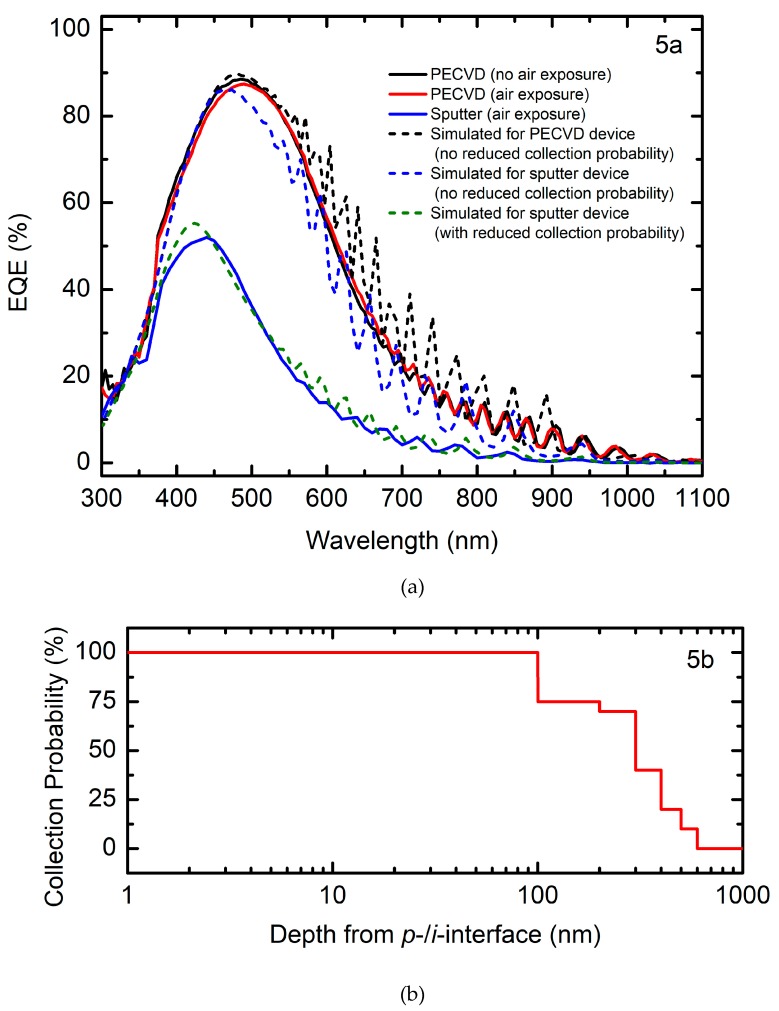
(**a**) External quantum efficiency (EQE) spectra for two *n-i-p* devices incorporating PECVD nc-Si:H absorbers, one of which is deposited without breaking vacuum (no air exposure, black solid line) and the other with vacuum breaks before and after intrinsic layer deposition (air exposure, red line) to simulate the processing of the samples with sputtered nc-Si:H absorbers (blue solid line). EQE spectra are simulated for a device with a PECVD *i*-layer (black dashed line), a device with a sputtered *i*-layer assuming complete carrier collection (blue dashed line), and a device with a sputtered *i*-layer with reduced collection probability (green dashed line). (**b**). Collection probability profile used in EQE simulation accounting for the recombination losses of photogenerated carriers within the 1 µm thick sputtered nc-Si:H *i*-layer.

Figure 5a shows EQE spectra over the range 300–1100 nm for the same three highest efficiency devices incorporating PECVD and RF magnetron sputtered nc-Si:H absorbers. There is little difference between either of the PECVD absorber device, both having nominally 1.8 µm thick absorbers. By contrast, the EQE spectra for a device with a 1µm thick magnetron sputtered nc-Si:H absorber is substantially lower for all measured wavelengths, more than that expected by the simple reduction in intrinsic layer thickness. *J_SC_* calculated from integration of EQE simulated using the optical response of PECVD nc-Si:H predicts a *J_SC_* reduction of about 4 mA/cm^2^ when the absorber layer thickness is decreased from 1.8 to 1 µm in *n-i-p* configuration devices. This indicates that the experimentally measured value of *J_SC_* = 6.2 mA/cm^2^ obtained for the device with the 1 µm thick sputtered absorber is much lower than the expected *J_SC_* for a similarly thick absorber made by PECVD. The optical response of sputtered [9] and PECVD nc-Si:H [4] are comparable and do not account for this additional discrepancy.

All these results indicate that the predominant limitation in devices with magnetron sputter deposited nc-Si:H absorbers stems from incomplete carrier collection originating from either or both recombination in the intrinsic bulk layer or at the interfaces with *n*- and *p*-type doped layers. Problems at the interfaces with the doped layers may arise from incompatibility of the two deposition processes for doped and undoped Si:H, PECVD, and sputtering. More energetic ion bombardment during sputtering of the *i*-layer on the PECVD *n*-layer may lead to surface damage. Similarly, the high *R* = 250 value for *p*-layer PECVD may lead to hydrogen etching of the underlying sputtered *i*-layer. The grain boundaries will etch preferentially over the crystalline grains, resulting in void rich and poorly passivated material at the interface. Additionally, depending on grain size and any porosity present between grains, exposure to laboratory air may have a greater impact on sputtered nc-Si:H relative to its PECVD counterpart. The decrease in EQE at all wavelengths indicative of incomplete carrier collection lowers *J_SC_* by 66% when comparing the highest efficiency devices with sputtered material to similarly processed PECVD material but with vacuum breaks. This reduction in current generated is greater than that of *V_OC_* and *FF* at 37% and 16%, respectively. As *FF* may be indicative of bulk *i*-layer performance [42,43,44] and *V_OC_* of the interfaces, results here suggest that recombination may be taking place predominantly at those interfaces inhibiting current collection. 

Simulations of EQE with a reduced carrier collection profile are used to further investigate the most likely sources of loss within the solar cell device incorporating the sputtered *i*-layer. The simulated EQE without collection losses (blue dashed line in 5a) for devices with a 1µm thick sputtered nc-Si:H absorber shows substantially higher values compared to measured EQE (blue solid line in 5a). This observed difference between simulated and measured EQE in the solar cell incorporating a sputtered *i*-layer can be attributed to incomplete carrier collection from portions of the *i*-layer. Modeling of the *i*-layer requires accounting for thickness variations in the relative amorphous and crystalline volume fractions and treating the PECVD and sputtered material slightly differently for agreement between simulation and measurement. The optical response of the 0.9 µm of the PECVD *i*-layers adjacent to the *p*-layers is described by those of crystal silicon [28], and the 0.9 µm adjacent to the *n*-layers is described by a Bruggeman effective medium approximation [27] of 0.9 volume fraction crystal silicon and 0.1 volume fraction 1.8 eV band gap a-Si:H [29]. This bilayer approach provides a simplified model for Si:H growth evolution with increasing crystallinity as a function of accumulated film thickness [4,7,8,9,10,44]. The thinner 1 µm thick sputtered *i*-layer is described as a bilayer of the same material properties although the crystal silicon component of the *i*-layer adjacent to the *p*-layer is 0.1 µm thick, while the effective medium approximation of crystalline silicon and a-Si:H near the *n*-layer remains the same thickness and volume fraction as for the PECVD *i*-layers. The sputtered *i*-layer is divided into ten 0.1 µm thick sublayers to introduce a collection probability profile to further improve agreement between simulated and measured EQE [45]. The collection probability profile is determined by fitting a variable fraction of carriers collected for each sublayer in a least squares regression to match the measured EQE. The resulting carrier collection probability profile as a function of depth from the *p*/*i* interface and simulated EQE spectrum incorporating that collection profile are shown in Figure 5a (green dashed line) and b. Some limitations of this approach are the simplification of the collection probability structure itself and the assumed absorber layer optical response which are expected to vary somewhat with deposition conditions [4,9,46,47,48] as well as thickness [3,47,48]. A more advanced optical modeling approach accounting for these simplifications would lead to improved agreement between the model and measured spectra, however the improved qualitative agreement already helps to discern the sources of collection losses. Namely, collection of photogenerated carriers decreases after 100 nm depth into the sputtered nc-Si:H *i*-layer reaching zero collection beyond 600 nm. Although the overall electronic quality of the sputtered nc-Si:H *i*-layer is likely lower than that prepared by PECVD, this reduction in collection probability indicates greater collection losses at the *n/i* interface possibly due to damage by increased ion bombardment of the *n*-layer during sputtering of the over-deposited *i*-layer. In contrast, complete carrier collection is observed near the *p/i* interface indicating that PECVD of the *p*-layer does not cause as substantial damage to the underlying sputtered *i*-layer. 

It should be noted that, regarding the relatively low efficiency of devices incorporating sputtered nc-Si:H absorbers, optimization of the material has been essentially limited to merely ensuring that the correct nanocrystalline phase is obtained. Variation of sputter deposition parameters would provide the potential to optimize the material further to achieve the best PV device performance. Material optimization principles and excursions in deposition parameter space similar to those explored for PECVD nc-Si:H can be adapted for optimization of sputtered nc-Si:H [49]. Fabrication of complete devices fully under vacuum would be expected to increase performance as well. Should incompatibilities exist between over-deposition by sputtering and PECVD, evaluation of sputtered doped layers [20] may also be considered. Thus, these results are best interpreted as a starting point upon which substantial improvements in device performance could reasonably be expected upon further material optimization.

## 4. Conclusions

Thin film nc-Si:H devices in the *n-i-p* substrate configuration have been fabricated with different intrinsic Si:H absorbers: standard PECVD nc-Si:H *i*-layer prepared entirely under vacuum, PECVD nc-Si:H *i*-layer where the sample is removed from vacuum between the deposition of the underlying *n*-layer and the overlying *p*-layer, and sputtered nc-Si:H *i*-layer with the same vacuum breaks. To our knowledge, this is the first report of a functioning PV device incorporating a sputtered nc-Si:H absorber layer. The devices with sputtered absorbers have low *PCE* in comparison to their counterparts incorporating PECVD absorbers. The vacuum breaks associated with the fabrication process of the sputtered-absorber device are identified to account for only ~1% absolute decrease in *PCE*. Further decreases in *PCE* for the sputtered absorber based devices are attributed to recombination losses in both the bulk and at interfaces. The collection probability profile obtained by comparing measured and simulated EQE for the device incorporating the sputtered nc-Si:H *i*-layer shows reduced carrier collection from partway through the bulk *i*-layer to the *n/i* interface indicating damage to the underlying *n*-layer during over-deposition of the sputtered nc-Si:H *i*-layer. These losses may be assessed and reduced by ensuring process compatibility and interfacial stability between deposition of the doped and undoped layers and performing all depositions in a load-locked system without unnecessary breaks from vacuum. The sputtered material itself also requires further optimization to reach performance levels comparable to those achievable with PECVD.

## Figures and Tables

**Table 1:** Deposition conditions for the individual layers in the nc-Si:H *n-i-p* solar cell configuration deposited on 15.24 cm × 15.24 cm soda lime glass (SLG) substrates in the load-lock cluster tool (i.e., with plasma enhanced chemical vapor deposition (PECVD) *i*-layers). The dopant source gases are each 5% dopant gas in H_2_ is by volume. “RT” denotes room temperature.

Layer	Substrate Temperature (°C)	Deposition Pressure (mTorr)	RF Plasma Power (W/cm^2^)	Gas Flow (SCCM)					
				Ar	5% O_2_ in Ar	SiH_4_	5% PH_3_ or B_2_H_6_ in H_2_	H_2_	R = H_2_/SiH_4_
Cr	RT	15	0.920	10	-	-	-	-	-
Ag	RT	15	0.920	10	-	-	-	-	-
ZnO	RT	5	0.920	10	-	-	-	-	-
n	200	1500	0.031	-	-	2	0.5 PH_3_	200	100
i	200	1500	0.043	-	-	5	-	125	25
p	100	1500	0.086	-	-	2	0.5 B_2_H_6_	500	250
ITO	150	4	0.582	10	3	-	-	-	-

**Table 2:** Comparison of device performance parameters of highest efficiency *n-i-p* cells incorporating nc-Si:H absorbers prepared with each absorber layer processing method.

Absorber i-Layer Preparation	*J_SC_* (mA/cm^2^)	*V_OC_* (V)	*FF* (%)	*PCE* (%)	*R_s_* (Ω cm^2^)	*R_sh_* (Ω cm^2^)
PECVD (no air exposure)	17.7	0.52	64.1	5.91	6.6	573.4
PECVD (air exposure)	18.5	0.50	54.2	5.08	7.3	574.1
Sputtered (air exposure)	6.2	0.32	45.6	0.92	15.4	445.6
